# Fabrication of a microfluidic chip based on the pure polypropylene material†

**DOI:** 10.1039/c7ra13334k

**Published:** 2018-02-27

**Authors:** Fupeng Liang, Yi Qiao, Mengqin Duan, An Ju, Na Lu, Junji Li, Jing Tu, Zuhong Lu

**Affiliations:** State Key Laboratory of Bioelectronics, School of Biological Science and Medical Engineering, Southeast University No. 2 Si Pai Lou Nanjing 210096 China zhlu@seu.edu.cn; Department of Bioengineering, Stanford University 443 Via Ortega Stanford CA 94305 USA

## Abstract

Polypropylene (PP) material has been widely used in the biomedical field for decades due to its high reliability in biochemical reactions. However, a pure PP material microfluidic chip has rarely been reported. Herein, a simple and rapid method has been developed to fabricate a pure PP microfluidic chip by a thermal bonding process using a PP film and PP substrate. An experiment for two-temperature PCR in the pure PP microfluidic system without a pre-treatment process has been successfully carried out. It is shown that the PP microfluidic chip has a high structural strength, tightness for water sealing, and low nonspecific adsorption, which promotes the practical application of microfluidics in the biomedical field. Compared to other existing microfluidic chip technologies, our pure PP material microfluidic chip and its fabrication method have many advantages such as high-speed production rate and extremely low cost. It can be achieved in industrial assembly lines for standardized manufacturing.

## Abstract 2

A robust pure polypropylene material microfluidic chip for the fast PCR and POCT applications has been achieved *via* low-cost, high-speed, and easy-standardized manufacturing.

## Graphical abstract

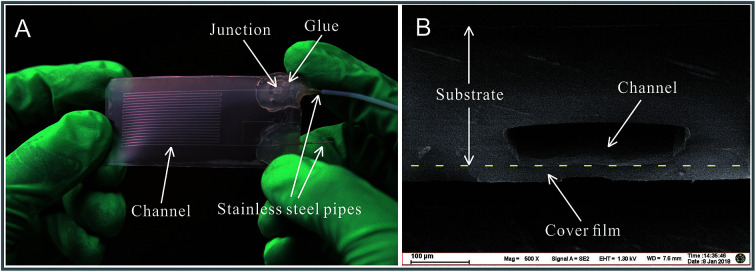

## Introduction

The soft-lithography^1,2^ technologies based on the polydimethylsiloxane (PDMS) material applied to the fabrication of a microfluidic chip are being developed for nearly 20 years, and PDMS is still the mainstream material for fabricating a microfluidic chip due to its simplicity in fabrication. However, the microfluidic technologies are just limited to labs and publications, and commercially available microfluidic products are scarce. An important reason for this is the serious defects in materials used for fabricating the microfluidic chip. Since PDMS is a porous material,^3^ the loss of reaction solution may occur, especially in the heating environment (such as polymerase chain reaction, PCR). A micro-channel or micro-container fabricated by the PDMS material is not suitable for long-time storage of solutions. Polymethylmethacrylate (PMMA) is an excellent polymer material that can be employed in the manufacture of microfluidic devices since it possesses excellent optical, thermal, and chemical properties.^4^ PMMA is not porous, but the glass transition temperature of the PMMA material is about 106 °C,^5^ which is not high enough for high-temperature sterilization (∼121 °C) in a biomedical process. Common organic chemical reagents, such as ethanol^6,7^ and ethyl acetate,^8^ may undergo reactions with the PMMA material; this limits the commercial applications (such as in reaction tubes) of the PMMA material in biomedical and other analytical chemistry fields. PMMA has poor resistance to many other chemicals due to its easily hydrolyzable ester groups. For example, PMMA can be hydrolyzed by alkali to produce acids and alcohols. As a result, PMMA cannot be used as reaction tubes in some cases such as for acridinium ester-based chemiluminescence^9–11^ detection that uses sodium hydroxide as the reaction reagent (acridinium ester-based chemiluminescence detection is an important approach in the medical diagnosis field). Therefore, the abovementioned obstacles make it difficult to establish an effective and reliable biological reaction system in microchannels of a PDMS or PMMA-based chip. It is important to establish new material platforms for practical medical microfluidic systems with high performance and reliability.

Polypropylene (PP) has many excellent properties such as high service temperature (up to 140 °C), high chemical resistance,^12^ low adsorption of biological macromolecules,^13^ non-toxicity, and good biocompatibility. The polypropylene material has been widely used in the biology and medicine fields such as in the manufacture of PCR reaction tubes and tips for biochemical experiment, reaction tubes for chemiluminescence immunoassay, and reagent tubes for biochemical analysis, syringes,^14–16^ and drug storage.^17^ In food fields, the polypropylene material is used for manufacturing microwave oven food boxes, baby bottles, and other daily necessities. Due to its excellent biocompatibility, the polypropylene material has even been used in medical grafts^18^ and as human implants.^19^ In literature, however, there is no practical case of pure polypropylene microfluidic chip fabrication although there exist several studies on the bonding processes between PDMS and polypropylene material in microfluidics fabrication field^20,21^ and few studies^5^ on the bonding processes between polypropylene and polypropylene material, in which the bonding temperature is 150 °C (lower than the melting temperature of the polypropylene material), leading to unreliable bonding. The difficult point in the fabrication of the polypropylene microfluidic chip is the bonding process between polypropylene materials due to its inert characteristics. In the injection moulding field, a significant interface^22^ was observed between the second shaped part and first shaped part in the product produced by a multiple injection moulding approach, which showed that the two parts did not integrate completely.

Herein, we proposed a novel pure polypropylene material microfluidic chip and its simple and rapid fabrication method. The microfluidic chip was fabricated by thermal bonding between a polypropylene sheet (used as the substrate) and a polypropylene film (used as the cover plate), and the polypropylene sheet was fully fused with the polypropylene film. We developed a special bonding method in which the interface between the polypropylene substrate and polypropylene cover plate could be rapidly melted and cooled afterwards. *Via* this method, a connection can be achieved between the two parts in a melting way (two pieces melting into one), and the structure of the microchannels can be protected from being destroyed. The chip has a high structural strength and stability. The fabricated polypropylene material microfluidic chip has low nonspecific adsorption, especially of DNA. Experiments, such as PCR, were successfully carried out to test the polypropylene microfluidic chips.

## Experimental

### Fabrication of the polypropylene microfluidic devices

**Fig. 1:**
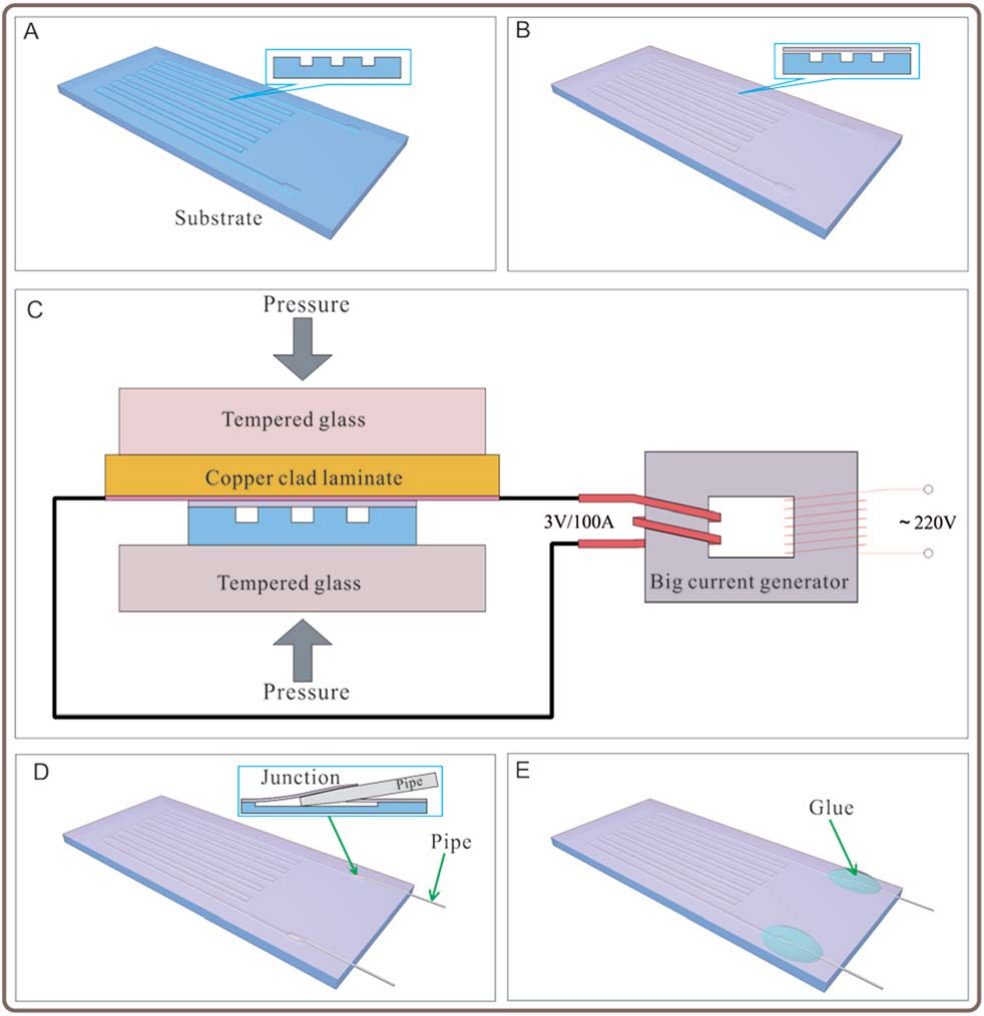
Schematic of the fabrication process of the pure polypropylene microfluidic chip. (A) Preparation of the polypropylene substrate with groove structures on the surface for constructing the microchannel. (B) Tiling of the polypropylene film on the polypropylene substrate. (C) Application of pressure on the polypropylene substrate and polypropylene film, and instant heating of the polypropylene film and the upper surface of the substrate by a high-power heater for a short period of time. (D) Use of stainless steel pipes to pierce through the polypropylene film to connect the microchannel inside the chip with outside. (E) Gluing of the stainless steel pipes on the polypropylene film to seal the junction.

The fabrication process of the polypropylene (PP) material microfluidic chip is shown in Fig. 1. In Fig. 1A, a polypropylene substrate was fabricated: the substrate with grooves could be fabricated by many approaches such as hot-pressing with a mould,^23^ micromachining,^24^ and injection moulding.^25,26^ Herein, we fabricated the polypropylene substrate by the hot-pressing method with a printed circuit board (PCB), which was used as a mould.^27^ The pattern on PCB was designed with circuit design software (Protel, Altium Corporation, Hobart, Australia) and customized from a printed circuit board factory, or engraved by the CNC (computerized numerical control) system. A copper film with designed shape was fabricated on the copper clad laminate, which was used as the mould. A polypropylene sheet with the thickness of 0.25 mm (purchased from Jincheng plastic materials Co. Ltd., Dongguan, Guangdong, China) was cut into a proper shape and placed on the patterned side of the PCB mould. Pressure was applied on the polypropylene sheet and the PCB mould with heating, and a negative pattern was formed on the polypropylene substrate. A polypropylene film with a thickness of 20 μm (purchased from Xiangfeng Co. Ltd., Henan, China) was tiled on the patterned polypropylene substrate to cover the surface of the substrate and seal the grooves in the substrate, as shown in Fig. 1B. Herein, the polypropylene film is biaxially oriented polypropylene (BOPP) film. As shown in Fig. 1C, the polypropylene film and the surface of the polypropylene substrate were heated to melt instantaneously to make the contacting parts of the polypropylene film and the polypropylene substrate bond together, and the non-contacting areas of the polypropylene substrate (such as the groove area) were not melted. A hot compression device containing a copper clad laminate used as the heating plate and a clamp with two flat tempered glass was built, as shown in Fig. 1C, which provided a stable and proper condition in the bonding process. The copper film of the copper clad laminate was tightly contacted with the polypropylene film with a pressure about 15–25 kPa, and the polypropylene film was heated by the copper film of the copper clad laminate, which was connected with a big current generator with an output voltage of 3 V and current of 100 A. The heating power density of the copper film was about 0.068 W per square millimetre. Duration of the current turn-on lasted for 7–8 s. The polypropylene substrate and film bonded together as the temperature ramped down, as shown in Fig. 1C and Video S1 in the ESI.† The channels of the chip were connected with outside by stainless steel pipes with an outer diameter of 0.3 mm and inner diameter of 0.1 mm. The stainless steel pipes with the pre-sharpened tips were pierced through the polypropylene film and entered into the microchannels to be used as an inlet and outlet of the microfluidic chip, as shown in Fig. 1D. The junctions between the stainless steel pipes and the polypropylene film were sealed with hot-melt adhesives to prevent leaking, as shown as Fig. 1E. Stainless steel pipes entered the interior of the microfluidic chip by puncturing the polypropylene film instead of cutting the holes on the polypropylene film in advance for mounting stainless steel pipes. Therefore, the stainless steel pipes were hooped tightly by the polypropylene film to prevent the hot-melt adhesive from flowing into the microfluidic chip before it cured.

In this method, the whole process of manufacture of the pure polypropylene microfluidic chip can be controlled within 3 minutes, including the process of fabrication of the polypropylene material substrate with grooves by the hot-pressing method. Compare to the other existing microfluidic chip technologies, our pure polypropylene material microfluidic chip and its fabricating method have many advantages such as high-speed production rate and extremely low cost. It could be achieved in industrial assembly lines for standardized manufacturing.

To verify the practicability of the pure polypropylene microfluidic chip fabricated by the process presented in this study, we conducted five tests: PCR experiment in the microchannel, droplet generation, DNA adsorption of the microchannel, strength tests, and leakage tests.

### Strength of the microchannel structure

Strength testing experiments were carried out for polypropylene chips. Herein, ten polypropylene chips are selected randomly and put through a pressure tolerance test, and the method is reported elsewhere.^28^ The outlets were sealed before pumping water from the inlets. Moreover, four polypropylene chips with a width of 25 mm were selected randomly to test the peel strength in a material testing machine (Model Instron 5943, Illinois Tool Works Inc., Norwood, MA, 02062-2643, US) by the approach of T-peel test.^29^ Length of the free-ends of the polypropylene film and polypropylene substrate were 30 mm.

### Leakage of the microfluidic chip

Leakage testing experiments were carried out as follows: 15 μl deionized water was stored in the microchannel of the polypropylene microfluidic chip, and the inlet and outlet were sealed up.

### Droplet generation

The polypropylene microfluidic chip was connected with two syringe pumps (Model L0107-2A, LongerPump Inc., Baoding, China), which pumped mineral oil (clear) and water (green) into two inlets of the chip separately. These two phases meet at a T-junction and form droplets. The velocity ratio of the oil phase and water phase is 3 : 1.

### DNA adsorption of the microchannel

To test the level of DNA adsorption of the polypropylene microfluidic chip, a new polypropylene microfluidic chip with its microchannel filled with the DNA-PicoGreen solution and deionized water sequentially was observed *via* a laser scanning confocal microscope system (Model Andor Revolution XD, Andor Technology Ltd, UK). Then, 5 minutes after pumping 5 ng μl^−1^ λ-DNA-PicoGreen solution into the chip, fluorescence images were obtained by the laser scanning confocal microscope system. After this, the chip was rinsed with deionized water and then analysed *via* the laser scanning confocal microscope system. The λ-DNA-PicoGreen solution is a mixture of 10 ng μl^−1^ λ-DNA and 2× PicoGreen solution of same volume. The reagents were obtained from the Quant-iT PicoGreen dsDNA Assay Kit (Life Technologies Corporation, USA).

### PCR experiment in the microchannel

**Fig. 2:**
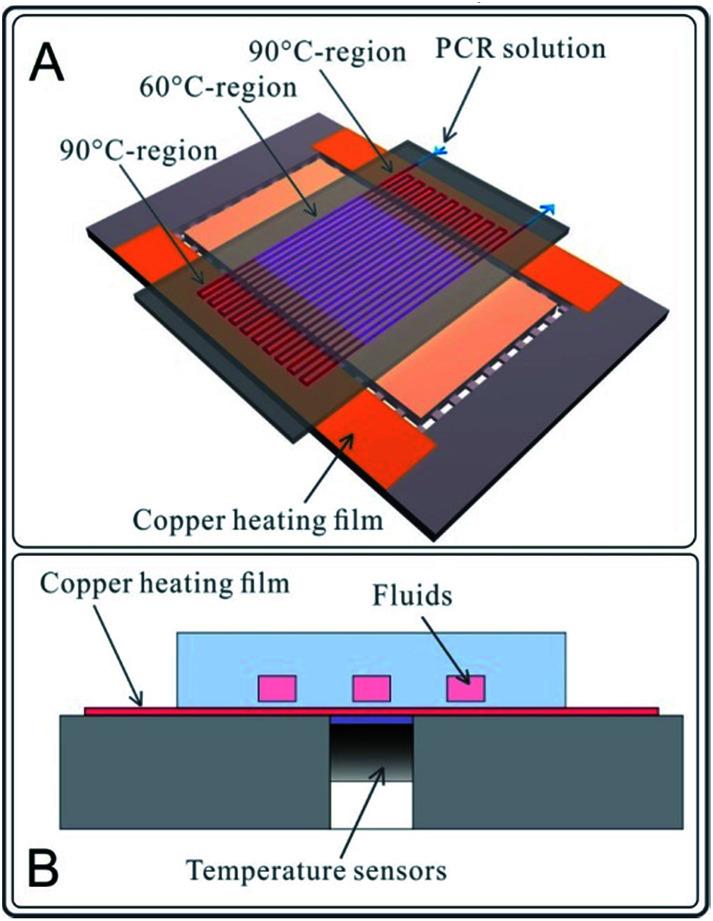
Two-temperature PCR experiments with the polypropylene microfluidic chip. (A) Schematic of the PCR experimental device. The polypropylene microfluidic chip was placed on a heating plate with 3 heating regions (90 °C–60 °C–90 °C) to perform 20 cycles of PCR reaction. (B) The profile of the PCR experimental facility. The heat generated by the copper heating film was rapidly conducted to the solution in the microchannel through the 20 μm thick polypropylene film (cover film). The temperature sensors mounted on the bottom surface of the copper heating film.

A two-temperature PCR experiment in the microchannel was designed and performed on the abovementioned chips. As shown in Fig. 2A, a two-part heating board was fabricated to provide two temperature zones (90 °C on the outer sides and 60 °C in the central part) for DNA amplification. PCR reagents were injected into a winding channel with the current velocity of 0.75 μl min^−1^ by a syringe pump (Model L0107-2A, LongerPump Inc., Baoding, China), as shown in Fig. 2A. The PCR had 20 cycles. The PCR reagent composition (20 μl system) is 10 μl SYBR mix, 1 μl template (K562 DNA ∼20 ng μl^−1^), 2 μl Primer, and 7 μl NF water. The polypropylene microfluidic chip used for the PCR reaction was completely new, and the microchannels of the polypropylene microfluidic chip were not pre-treated (only rinsed with deionized water) before the PCR reaction. Reactions with same reagents were performed using a common traditional thermal cycler (PCR apparatus, Model 9902, Life Technologies Corporation, USA). After PCR, the electrophoresis processes for 10 μl production of chip-PCR and 10 μl production of common PCR were performed.

The heat generated by the copper film was conducted rapidly to the liquid in the microchannels through the polypropylene film (cover film) of the polypropylene microfluidic chip, as shown in Fig. 2B. Temperature sensors were mounted on the bottom side of the copper film. The heating process was controlled by the control circuit in real time.

## Results and discussion

### Obtaining the polypropylene microfluidic chip

**Fig. 3:**
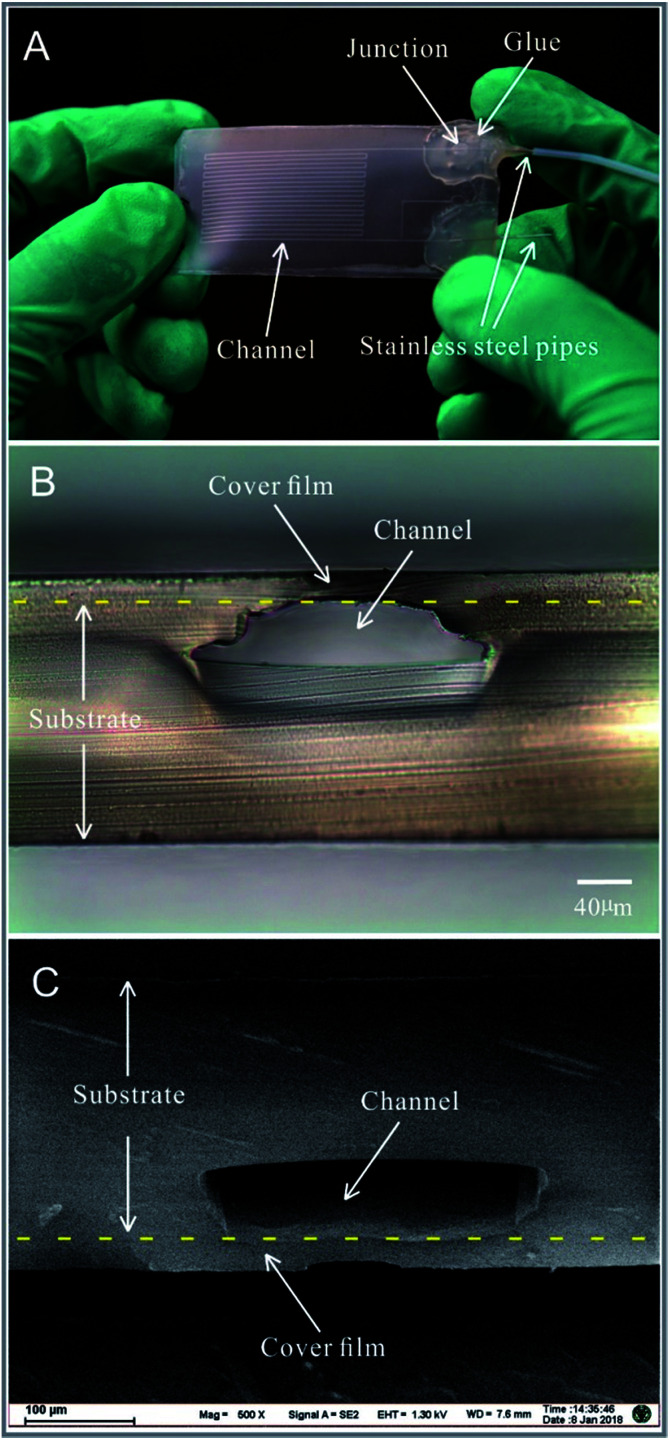
Images of the pure polypropylene microfluidic chip: (A) An image of the microfluidic chip with stainless steel pipes and microchannels obtained at the angle of ∼90° between the incident light (illuminating the microfluidic chip) and the optical axis of the camera lens. (B) The image of the cross-section of the microchannel obtained by an optical microscope. (C) An SEM image of the microchannel's cross-section. The cross-sections were obtained by blade cutting.

A pure polypropylene microfluidic chip is shown in Fig. 3A. Stainless steel pipes with an outer diameter of 0.3 mm and inner diameter of 0.1 mm were mounted on the microfluidic chip as an inlet and outlet. A hot-melt adhesive was applied on the junction between the stainless steel pipes and the polypropylene film to fix the stainless steel pipes and prevent leakage of the liquid. The cross-sections of the polypropylene microfluidic chip are shown in Fig. 3B and C and Fig. S1 in the ESI.† The microchannel's cross-sections were observed *via* an optical inverted microscope (Model IX2-UCB, Olympus Corp, Japan) with a CCD camera (Model GO-3-CLR-10, QImaging Corporation, Canada) and a field emission scanning electron microscope (Model Ultra Plus, Carl Zeiss Microscopy GmbH, Germany). In the bonding process, the polypropylene film and the upper surface of the polypropylene substrate were heated, melted, and squeezed. The region of the microchannels close to the polypropylene film was deformed, which only arched the structure of the microchannels without influencing the functions. The groove structures on the polypropylene sheet were also squeezed by the mould in the molten state (during the process of fabrication of the polypropylene substrate); this made the density of the polypropylene substrate inhomogeneous and the crystal structure of the polypropylene substrate change (which could be seen with different refractive indices in the cross-section of the polypropylene substrate observed *via* an optical inverted microscope in Fig. 3B). The images of the microchannel's cross-sections indicated that the polypropylene cover film and polypropylene substrate were melted into one, as shown in Fig. 3B and C.

### Bonding process

**Fig. 4:**
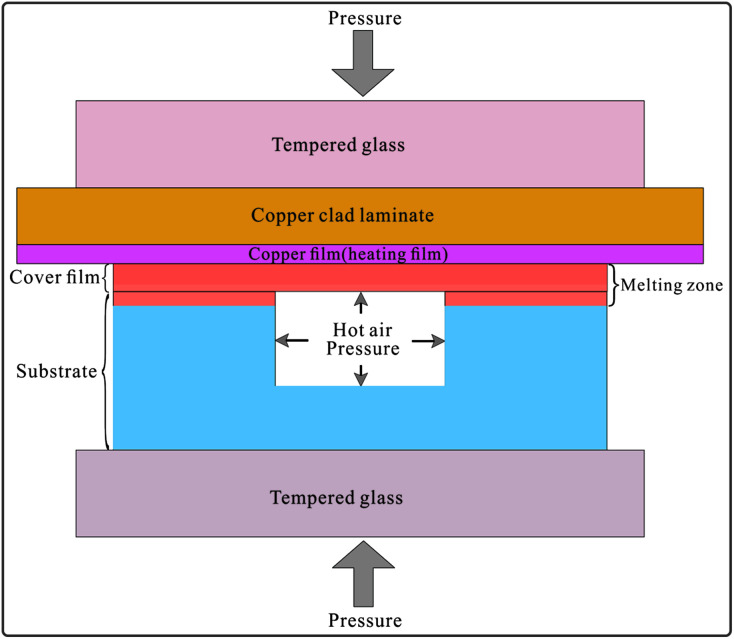
The principle of the bonding process. The polypropylene film (cover film) and the upper surface of the polypropylene substrate were rapidly heated to the melting state by the copper film (copper foil) of the copper clad laminate and then cooled down fast when the current was cut off. The air sealed in the grooves generated an outward pressure when it was heated. The outward expansion pressure could prevent the microchannel from collapsing during the bonding process.

The bonding principle is shown in Fig. 4. The copper film with the thickness of 50 μm and the area of 100 mm × 44 mm on the copper clad laminate was used for high-power heating. The polypropylene film (cover film) with the thickness of 20 μm could be heated to melt instantaneously and then conducted heat to the upper surface of the polypropylene substrate. Both formed the melting zone, as shown in Fig. 4, and fused together to form the bonding structure. During the fusion process, the air sealed in the grooves was heated to expand and generate an outward pressure. The outward pressure prevented the melting polypropylene material from moving into the grooves, thus avoiding the collapse of the microchannels. A large surface/volume ratio (the thickness of the copper film is 50 μm, and its surface area is 100 mm × 44 mm) and high thermal conductivity make the heating or cooling of the copper film quite rapid. After the applied current was turned off, the copper film cooled down rapidly. The melting zone quickly turned into the solid state; this avoided excessive heating and shortened the melting time to protect the groove structures from collapsing.

**Fig. 5:**
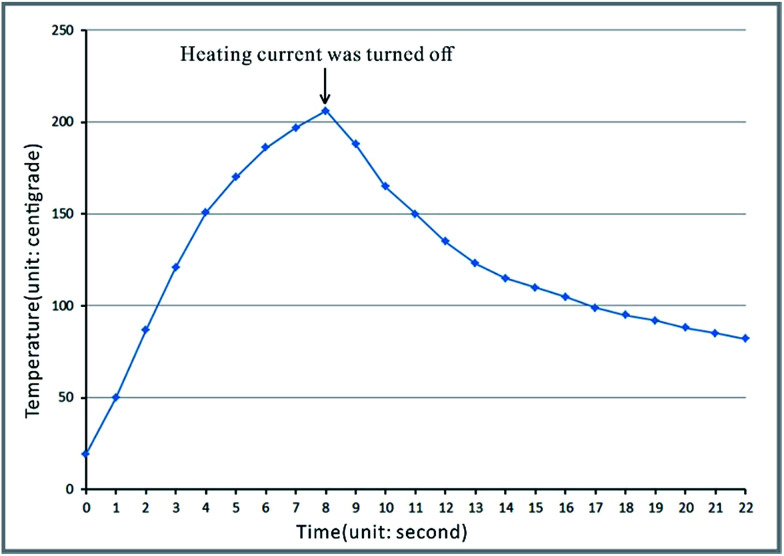
The temperature curve of the copper film on the copper clad laminates used as heating plates in the bonding process.

In the process of heating, the temperature higher than 165 °C lasted for about 5 seconds, *i.e.* about 3 seconds before cutting off the heating current and about 2 seconds after cutting off, as shown in Fig. 5. The variation of temperature is shown in Video S4 in the ESI.† The temperature was determined by the temperature sensor stuck to the copper film (heating film) on the opposite side of the polypropylene film, as shown in Fig. S2 in the ESI.†

The melting time and cooling speed are the key factors in the bonding process. Bonding of the polypropylene substrate and the polypropylene film with a long period heating will lead to its failure. If the polypropylene substrate and the polypropylene film are melted by general heating means, the substrate would become soft and flowable, which would result in the collapse of microchannels under the action of pressing. On the other hand, if the polypropylene substrate and the polypropylene film do not melt, such as the bonding approach mentioned in the literature,^5^ the microchannels will not collapse, but the binding force between the polypropylene substrate and the polypropylene film would be low, which would result in automatic spalling at the interface between the polypropylene film and the polypropylene substrate. The regular thermal bonding technique that is usually used for the fabrication of plastic microchips^30^ (*i.e.* PMMA, PC) is not suitable for PP because the bonding temperature is below the melting temperature.

Polypropylene is hard and not absolutely smooth in micro-meter scale after being produced (there are scratches, protrusions, depressions, and adsorption particles). Compared to PDMS (the mainstream material for fabricating the microfluidic chip), the microchannels of pure polypropylene material could not be fabricated by pre-surface-treated bonding like PDMS.^20^ However, in the present melting bonding process, the soft melting polypropylene can deform and flow under pressure. The residual air between the polypropylene film and the polypropylene substrate (except the air in the grooves) is driven away. The defects (such as scratches, bumps, depression *etc.*) on the surface of the polypropylene substrate are eliminated, and the particles or dust on the surface of the polypropylene substrate are wrapped up. As shown in Video S2 in the ESI,† the scratch defects of micron-width in the background observed using an optical microscope are on the bottom surface of the polypropylene substrate (the upper surface of the polypropylene substrate is bonded with the polypropylene film). The scratch defects on the upper surface of the polypropylene substrate were eliminated in the bonding process. All scratch defects originated from the polypropylene sheet production process, during transportation, or in the fabrication process of the microfluidic chip do not affect the function and the bonding process of the microfluidic chip.

### Transmittance of the microfluidic chip

The pure polypropylene microfluidic chip is suitable for optical detection in conventional biomedicine fields since polypropylene is optically transparent with a transmittance of about 60% for a 0.25 mm thick sheet (the transparency of polypropylene sheets purchased from different manufacturers may be different). Although transmittance reduces when the polypropylene sheet is being squeezed and melted in the process of hot-pressing, the fluidic behaviour in microchannels can still be observed or detected from the side of the polypropylene film by an optical microscope due to its high transmittance (about 95% for the thickness of 20 μm). After bonding, the transmittance of the non-channel region of the polypropylene chip was about 50%, whereas that of the polypropylene film was about 92%. The transmittance can be competent for common optical detection, see the Video S2† in the ESI.† Thus, the current fabrication method does not limit the thickness of the polypropylene sheet used for the substrate.

### Strength of the microchannel structure

In the pressure tolerance test,^28^ all ten chips survived at a 0.2 MPa pressure, whereas some survived under 0.55 MPa (most of the leaking occurred at the inlets). In the T-peel test^29^ with four chips, all the bonding areas between the polypropylene films and polypropylene substrates could not be broken before the polypropylene films fractured themselves. The fracture force was between 45 N and 53 N, as shown in Fig. S3 in the ESI.† Reliability of the polypropylene chips satisfies the requirement for their normal use in microfluidics research. The pure polypropylene microfluidic chips have no problems of adhesion abating, which are commonly observed in PDMS-based microfluidic chips and PMMA-based microfluidic chips, between the polypropylene substrate and polypropylene film after long-time storage. The structural strength of the pure polypropylene microfluidic chips meets the requirement of ordinary microfluidics applications.

### Leakage of the microfluidic chip

The total weight of the microfluidic chip and water sealed inside the chip remained constant after storage for 6 months in atmospheric environment, where the volume of the water remained unchanged. The polypropylene microfluidic chip with a 20 μm-thick cover film has an excellent water tightness.

### Droplet generation

Uniform droplets were generated (as shown in Video S2 in the ESI†). Droplet generation is one of the common applications of microfluidics. The polypropylene microfluidic chips are competent for it.

### DNA adsorption of the microchannel

**Fig. 6:**
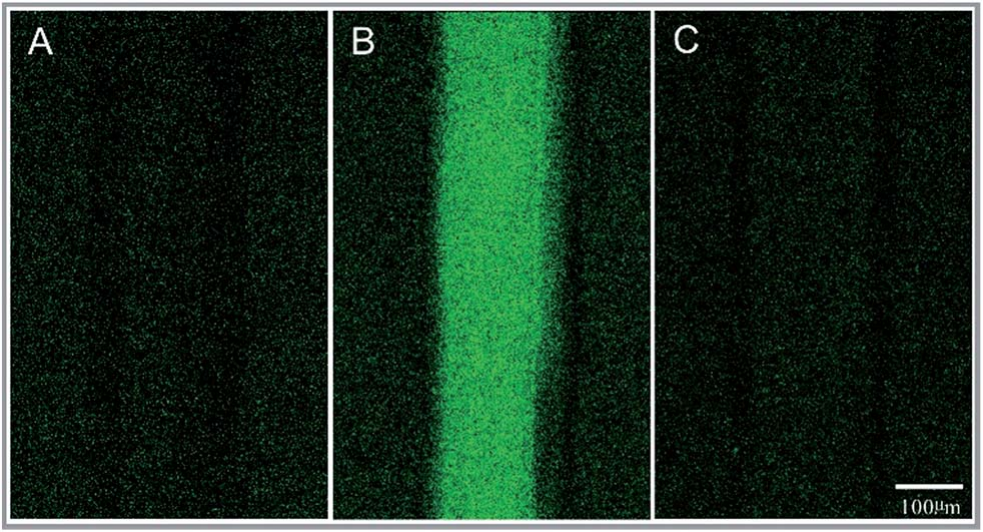
Observation of the 5 ng μl^−1^ aqueous solution of the fluorescence-labelled DNA in the microchannel using a laser scanning confocal microscope system. (A) The microchannel with deionized water, before being filled with a 5 ng μl^−1^ aqueous solution of fluorescence-labelled DNA. (B) Filling with a 5 ng μl^−1^ aqueous solution of fluorescence labelled DNA for 5 min. (C) After rinsing.

The result showed that the fluorescence intensity of the microchannel was the same as that of the non-channel area (background); this indicated that the nonspecific adsorption of DNA in the polypropylene microchannel was low, as shown in Fig. 6.

### PCR experiment in the microchannel

**Fig. 7:**
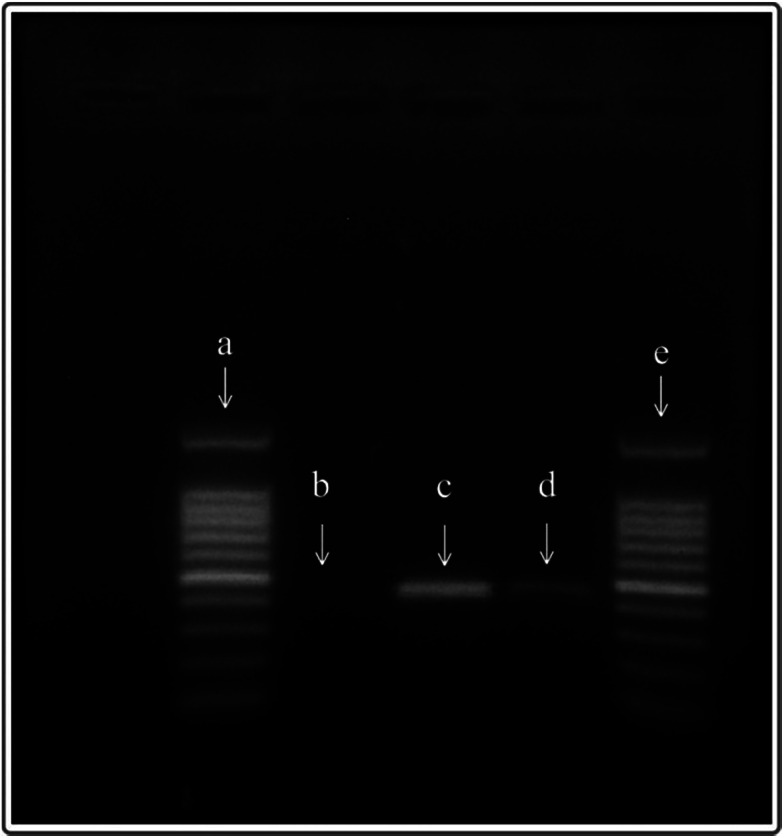
The electrophoresis results of PCR products, (a and e) the 100 bp DNA marker, (b) the negative control group, (c) the products amplified by a common thermal cycler (PCR apparatus), and (d) the products amplified in the polypropylene microfluidic chip.

PCR reactions could be performed effectively in the polypropylene microfluidic chip (Fig. 7), but the efficiency of the reaction was lower than that of the ordinary PCR. Although the PCR efficiency based on the polypropylene microfluidic chip is weaker than that of the ordinary PCR instrument in the present experiments, it can be improved by optimizing the reaction conditions and parameters of the PCR in the microfluidic chip.

## Conclusions

Polypropylene material has been widely used in the biomedical field for decades. The reliability of the polypropylene material in biochemical reactions has been fully verified. We have successfully fabricated pure polypropylene material microfluidic chips by thermally bonding the polypropylene cover film and polypropylene substrate. Unlike PDMS or PMMA-based microfluidic systems, the polypropylene microfluidic chip has a high structural strength due to its fused bonding. The present studies would bring the excellent performances of the polypropylene material to microfluidics, especially for a biological reaction system in microfluidic chips without pre-treatment, and stable and reliable storage of reaction reagents in microfluidic chips, which has prospects for solving the obstacles, such as low reliability, high cost, and low usability, in the present microfluidics field and would promote the microfluidics technologies to be widely used in the biomedical field, particularly in point of care testing (POCT). The fabrication method is simple and extremely low cost and has a high production rate. It could be achieved in industrial assembly lines for standardized large production manufacturing.

## Author contributions

The manuscript was written through contributions of all authors. All authors have given approval to the final version of the manuscript.

## Conflicts of interest

There are no conflicts to declare.

## Supplementary Material

RA-008-C7RA13334K-s001

RA-008-C7RA13334K-s002

RA-008-C7RA13334K-s003

RA-008-C7RA13334K-s004

RA-008-C7RA13334K-s005
