# Supplementary material for: Fabrication of a microfluidic chip based on the pure polypropylene material
Source: RSC Adv. 2018 Feb 27;8(16):8732–8. doi: 10.1039/c7ra13334k (PMC9078621; doi:10.1039/c7ra13334k)
Supplement: RA-008-C7RA13334K-s001 [file RA-008-C7RA13334K-s001.pdf]

Electronic Supplementary Material (ESI) for RSC Advances.
This journal is © The Royal Society of Chemistry 2018

Supporting Information

Part Ⅰ. Video files

Video S1. Bonding process in fabrication of pure polypropylene material microfluidic chip. The chip can be bended due to the stress generated in the bonding process as seen in the Video. Using a thicker substrate can greatly reduce the chip bending (such as, 2 mm thick substrate). Also, we can eliminate or correct the chip bending by clamping apparatus (chip holder).

Video S2. Droplet generation in the pure-polypropylene material microfluidic chip. The process was observed under optical inverted microscope.

Video S3. The microchannel filled with 5ng/μl aqueous solution of fluorescence labeled DNA. After rinsed with deionized water, the fluorescence intensity of the microchannel was same as the non-channel area(background),which indicated the nonspecific adsorption of DNA in the polypropylene microchannel was low. The process was observed under laser scanning confocal microscope system.

Video S4. The in-situ temperature measurement during the bonding process.

Part Ⅱ. Pictures

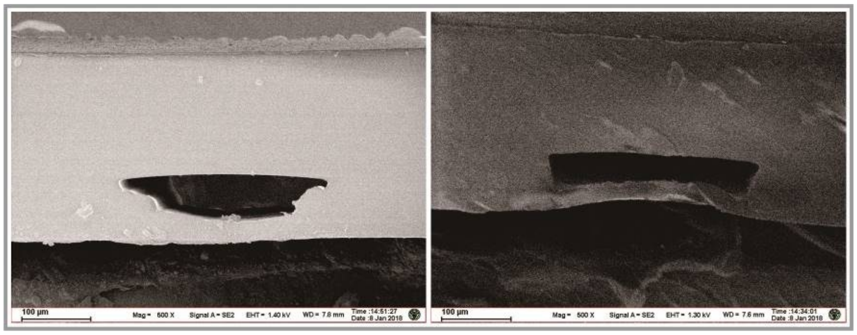

Fig. S1. SEM pictures of the microchannel's cross-section. The cross-sections were obtained by blade cutting.

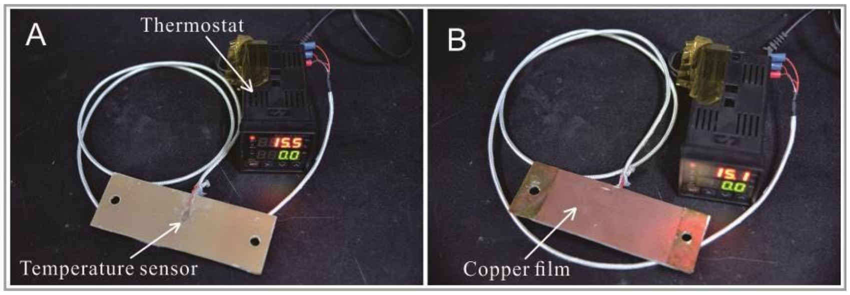

Fig. S2. A temperature sensor stuck to the copper film (heating film) of the copper clad laminate.

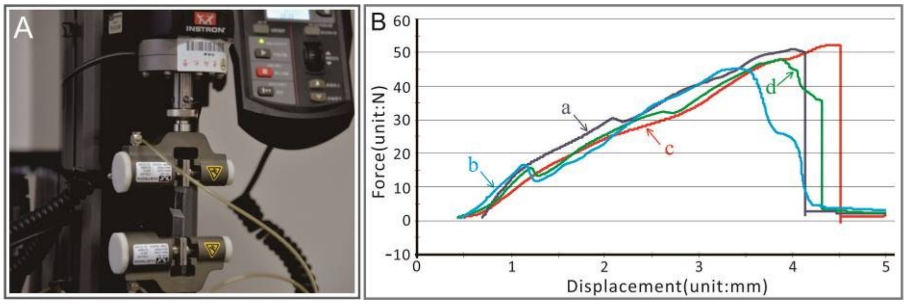

Fig. S3. The peel strength test for the polypropylene chips with width of 25mm in a material testing machine. (A) The material testing machine and the polypropylene chip. (B) Force curves for 4 different polypropylene chips.

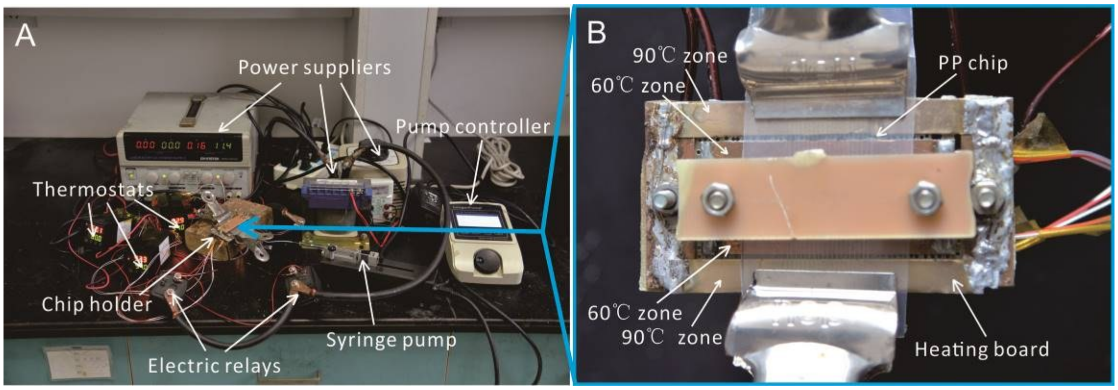

Fig. S4. The microfluidic PCR experimental platform. (A) The whole platform, including 3 power suppliers, 3 temperature controllers(thermostats), chip holder, syringe pump and its controller, various electric connecting wires, and so on. (B) Chip holder, including heating board with 2 temperature zones, microfluidic chip and chip clamping apparatus.
